# Automatic non-symbolic numerosity processing in preschoolers

**DOI:** 10.1371/journal.pone.0178396

**Published:** 2017-06-23

**Authors:** Xiaoshuang Zhu, Yinghe Chen, Yanjun Li, Zhijun Deng

**Affiliations:** Institute of Developmental Psychology, Faculty of Psychology, Beijing Normal University, Beijing, P. R. China; University of Bath, UNITED KINGDOM

## Abstract

There has recently been an increasing focus on the development of automatic processing of numerical magnitude. However, little effort has been made to explore automatic access to non-symbolic numerical magnitude in preschool children. In experiment 1, we used a non-symbolic physical size comparison task in 3- to 6-year-olds to examine developmental changes and the effect of ratio and counting principle knowledge. Results showed that the existence of automatic non-symbolic numerical processing began at age 3–4 years and size congruity effects tended to reduce with increasing age from 4 years old. The study also found that non-counting-principle knowers had a larger congruity effect, and in low ratio conditions the size congruity effect was more easily found. In addition, symbolic number comparison ability was negatively related to size congruity effect. In experiment 2, we explored the relationship between inhibition skill and size congruity effects, as well as interference and facilitatory components in children aged 4 years old. Results showed no correlation between inhibition skills and the size congruity effect and only interference effects were found. We also found a larger interference effect in low ratio conditions than in high ratio conditions.

## Introduction

Numerosity is an important and useful abstract dimension in our lives. In recent decades there has been a growing amount of research into the developmental trajectories of automatic processing of numerical magnitude [1–4]. An example of this is automatic processing shown by the size congruity effect, measured by the Size Congruity Task [5–7]. In this paradigm, participants must choose the physically larger number or dots and ignore a task-irrelevant dimension (the numerical magnitude). The tasks contains a congruent condition (physically larger is numerically larger), a neutral condition (the number is the same but the physical size differs) and an incongruent condition (physically larger is numerically smaller). The reaction times for congruent trials were shorter than incongruent trials and the accuracy of congruent trials was higher than incongruent trials. The size congruity effect suggested that perceptual processing was influenced by the quantity information, which was processed without conscious guidance and awareness. Tzelgov, Henik, Sneg, and Baruch (1996) suggested that a process is automatic if it is not part of the task requirements [8]. Consequently, these results in the size congruity task have demonstrated that automatic processing of numerical magnitude (task-irrelevant dimension) occurs. The majority of studies of automaticity in numerical magnitude processing have focused on symbolic stimuli and adult subjects [6, 9, 10, 11]. Whilst a series of studies has found similar effects in children, significant developmental changes in the automaticity of numerical magnitude processing also exist [5, 7, 12, 13, 14, 15].

Children of early kindergarten age rarely use number symbols. They may be able to recognize numbers, but do not have automatic access to those number symbols yet [1, 5, 12]. Thus, the ability to automatically process non-symbolic numerosities is more important for them; however, few studies have given attention to the automatic processing of magnitude in children using non-symbolic stimuli. Rousselle and Noel (2008) creatively used an original non-symbolic numerical Stroop paradigm to examine developmental changes in the automatic processing of numerosity in 3-, 4-, 5- and 6-year-olds [14]. Subjects had to compare the total filled areas of collections of dots or bars between 2 pictures. In congruent trials, the collection that was larger in number also had the larger total filled area, contrary to the incongruent trials. They found that the existence of interference between numerical and perceptual information began at age 3 and the congruity effect tended to increase with age. However, other researchers have found different patterns of the development of automatization of numerosity. Gebuis, Kadosh, and Haan (2009) used another non-symbolic Stroop task in 5-year-old children. In this task, children were asked to judge only the physical size of dots. The results demonstrated that a larger size congruity effect existed in 5-year-olds than in adults [1]. Gebuis et al. (2009) used task and event-related potentials (ERPs) as a measure to investigate the developmental changes of automatic numerosity processing in children between 6 and 8 years, and adults [13]. Both the behavioral and ERP data showed that the non-symbolic size congruity effect decreased with increasing age. However, results have been inconsistent, necessitating the further study of developmental change in the automatic processing of numerosity.

The development of automatic access to numerosity may be related to the frequency of utilization of non-symbolic or symbolic magnitude information. Some researchers believe that children, without knowing symbolic numbers, mostly make use of non-symbolic notation [1], and as soon as symbolic notation is mastered, they will predominantly use a symbolic number, allowing more precise calculation. Learning symbolic numbers leads to a lack of continued training and decreased utilization of non-symbolic numerosity processing, which eventually may result in a reduction of non-symbolic automaticity [13]. Therefore, the grasp of new symbolic numbers may have a negative relationship with the development of automatic processing of non-symbolic numerosity. The mastery of counting principles (CPs) is regarded as a crucial and important step in the development of a symbolic number system [16–18]. CP knowledge may be an effect factor in the automatic processing of non-symbolic numerosity in young children.

The approximate number system (ANS) is a phylogenetic and ontogenetic system that represents large, approximate numerical magnitudes without the need to count or rely on numerical symbols [19]. Many studies have found that people rely on the ANS in intentional processing of large numerosity [19, 20, 21]. This gives rise to a question of whether the ANS is related to the automatic processing of numerosity in early childhood. The ‘‘ratio effect” is the main signature of the analogue number system [19]. When asked to compare the relative magnitudes of 2 numbers or sets, when the ratio between the 2 numbers being compared is lower (or the distance is larger), participants are faster and more accurate, in accordance with Weber’s Law; this is known as the numerical ratio (distance) effect [20, 21]. There is a similar effect in the automatic processing of numerosity. Some studies have found that ratio had an influence on the automatic processing of symbolic and non-symbolic magnitude, with a larger congruity effect in the lower ratio or larger distance condition in 6- to 8-year-old children [6, 13, 22, 23]. Whether young children use approximate number representation when performing the non-symbolic numerical Stroop task, is worthy of further exploration.

In our study, we used the non-symbolic physical size comparisons paradigm in 3- to 6-year-old children to explore the developmental characteristics of automatic processing. To investigate the role of the ANS, we controlled the effect of ratio between the compared sets. We used a number comparison task that could measure symbolic number processing abilities, and selected children who had not yet acquired counting principles, to study the influence of mathematical language in the development of non-symbolic automatic processing.

## Experiment 1

### Methods

#### Ethics statement

This research was approved by the local ethical committee of Beijing Normal University. We obtained informed written consent from the next of kin, caretakers, or guardians on behalf of the minor/child participants involved in the study, according to the institutional guidelines of Beijing Normal University.

#### Participants

A total of 80 children participated in the study (mean age ± SD: 54.91 ± 11.47 months; range: 39–75 months; 34 boys), all from one kindergarten in Beijing, China. An additional 6 children were tested but were excluded as they did not complete the tasks. The kindergarten had 3 grades: Junior Class (34 participants; mean age ± SD: 43.09 ± 2.64 months, range: 39–48 months, 15 boys), Middle Class (23 participants, mean age ± SD: 58.23 ± 2.59 months, range: 52–60 months, 11 boys) and Senior Class (23 participants, mean age ± SD: 69.09 ± 4.21 months, range: 62–75 months, 8 boys). To test the effect of age, we also grouped the children into a 3-year-old group (*n* = 15, mean age ± SD: 40.71 ± 0.88 months, range: 39–42 months, 8 boys), a 4-year-old group (*n* = 23, mean age ± SD: 46.38 ± 3.64 months, range: 43–54 months, 7 boys), a 5-year-old group (*n* = 27, mean age ± SD: 60.80 ± 2.63 months, range: 57–66 months, 15 boys), and a 6-year-old group (*n* = 15, mean age ± SD: 71.60 ± 2.60 months, range: 67–75 months, 4 boys).

#### Apparatus

E-Prime 1.1 software installed on a 17-inch Dell computer was used to program the presentation of stimuli and to collect correct rate and reaction time (RT) data.

#### Stimuli and materials

**Fig 1:**
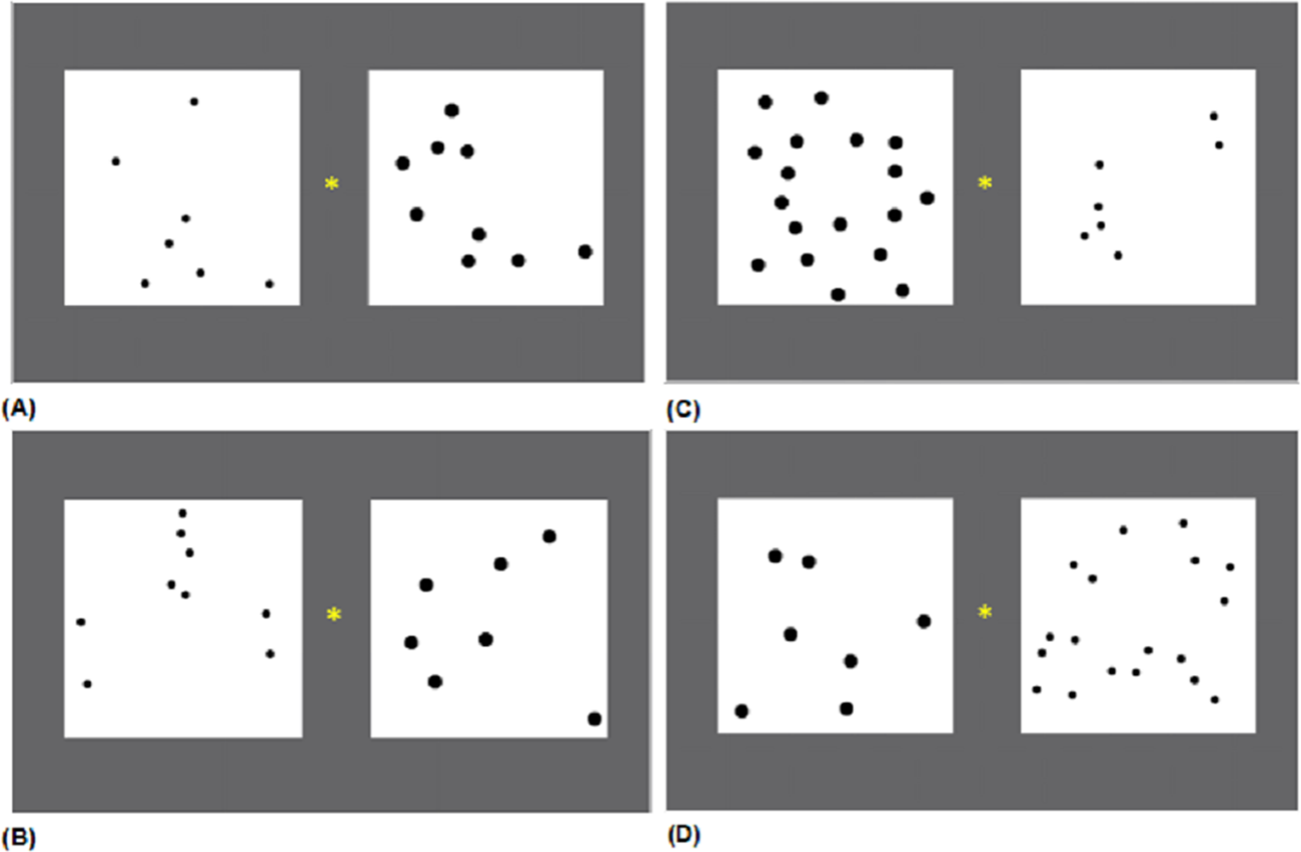
Examples of stimuli presented in a non-symbolic physical size comparisons task. A. Congruent pair, 7 vs. 9 dots (high ratio), both numerically and physically larger compared with numerically and physically smaller dots which together have a smaller total surface area. B. Incongruent pair, 7 vs. 9 dots (high ratio), numerically larger but physically smaller dots compared with numerically smaller but physically larger dots which together have a larger total surface area. C. Congruent pair, 7 vs. 18 dots (low ratio). D. Incongruent pair, 7 vs. 18 dots (low ratio).

**Non-symbolic physical size comparisons task/size congruity task**. To measure the ability of the children to automatically process numerical magnitude, a non-symbolic physical size comparison task [1] was administered. Participants were presented with 2 groups of black dots on the computer screen. The size of dots in one group was larger (radius: 0.9 cm) than the other (radius: 0.5 cm). To control total contour length, we used Microsoft Visual C++ 6.0 to generate 2 dot sets automatically with the contour length of one equal to the other. Both groups of dots were randomly distributed on a white square (9 cm × 9 cm; Fig 1). The physically smaller dots set had a smaller total surface area to avoid conflict between visual properties. Participants were asked to choose the physically larger group as fast as they could without making any errors. A total of 56 trials were administered to each participant: 8 practice trials, 24 incongruent and 24 congruent trials (Fig 1). The larger dots appeared equally on both sides of the computer screen. The number of dots in each group varied from 6 to 36, the pairs were matched in high (0.78~0.89, 12 incongruent and 12 congruent trials) and low (0.37~0.39, 12 incongruent and 12 congruent trials) ratios (see S1 Table).

**Color discrimination task**. To measure the basic speed of processing condition, we used a color discrimination task. Participants had to press a button when a green square was present on the grey background. Twenty trials were randomly displayed, half of which presented the green square. Before the formal test, 4 practice trials were completed. RT was recorded.

**Give-a-Number task**. The experimenter placed 20 black chess pieces on the table in front of the child. The child was then asked to put a particular number of chess pieces on a plate. The number of the chess pieces requested started from 1, followed by the numbers from 2 to 9 in an irregular order (never consistently ascending or descending), to avoid children reciting numbers sequentially. If the children gave a wrong answer, the experimenter asked for the number below the failed number and then asked for the failed number again. The task ended with the number 9 or with 2 incorrect answers out of 3 for a given number. In previous studies using this task [16, 24], children who succeed in giving 5 or more chess pieces were assigned to a counting-principle-knowers (CP knowers) group (*n* = 17, mean age: 42.48 ± 2.27 months, range: 39–47 months, 8 boys), and the remaining children assigned to a non-CP-knowers group (*n* = 17, mean age: 43.49 ± 3.11 months, range: 40–48 months, 7 boys). Our study participants were tested with this task, but only the children in Junior Class were divided into CP-knower and non-CP-knower groups. The children in the other grades were CP knowers.

**Number comparison task**. We used a number comparison task to measure the symbolic number processing abilities of the children. All the children participated in a number naming task, while only children who could pass the number naming task participated in this task (Number of children who passed in each age group: Age 3: 0; Age 4: 5; Age 5: 24; Age 6: 14). In the number naming task, there were 2 disordered sequences of number ranging from 1 to 9, for example, 2, 6, 4, 5, 9, 1, 8, 3 and 6. Children were asked to name numbers of two disordered sequences respectively. If children could answer both sequences correctly, they would pass the task. A total of 43 children passed this task and completed the number comparison task. In the number comparison task, 2 single digit numbers (ranging from 1 to 9) were presented on a computer screen, and the participants were asked to choose the numerically larger number as fast as they could without errors. Both numbers were of the same size. The correct answer appeared equally on both left and right sides of the trials. There were 60 trials, including 6 practice trials. The ratio between the 2 numbers varied from 0.11 to 0.89 (27 levels altogether) to control the influence of ratio, and each ratio was repeated twice in random order [6].

#### Procedure

Participants were tested individually in a quiet room. First, the children completed the Give-a-Number task. The experimenter then put a 12-color card with different color squares on the table before the children, and asked them to choose the green, blue, and gray color. When they completed this correctly the color discrimination task was administered. Rest time between each task was approximately 2 minutes. Finally, the children completed the non-symbolic physical size comparison task and number comparison task. Half of participants completed the number comparison task first. The rest time between these 2 tasks was approximately 2 minutes. The entire procedure lasted approximately 25–30 minutes.

### Results

#### Counting principle knowledge effect

**Fig 2:**
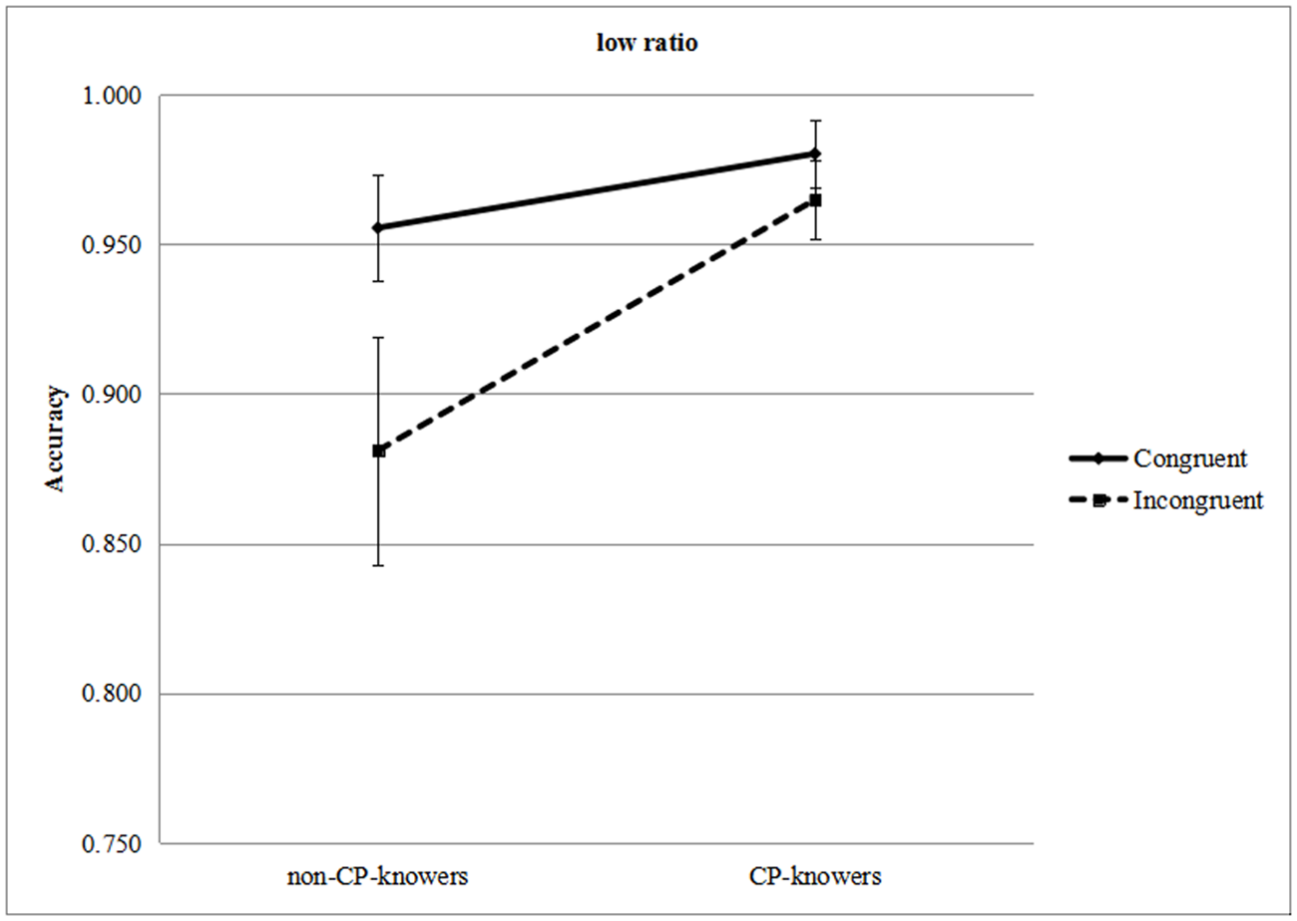
Accuracy results of interaction of CP knowledge and condition in low ratio.(Error bars showed the standard errors).

**Accuracy analyses**. We selected the 34 Junior Class children to analyze the CP knowledge effect. The accuracy of the trials reached 94.9%. A 2 (CP knowledge: CP knowers or non-CP knowers) × 2 (ratio: high ratio, low ratio) × 2 (condition: congruent, incongruent) analysis of variance (ANCOVA), with repeated measures for ratio and condition, controlling for months of age, was run on the proportions of correct answers. We used Mauchly’s test for sphericity and the Box's M test for homogeneity of variance (Box's M = 30.076, *F* = 2.597, *p* = 0.004). When the tests were significant we applied a Greenhouse—Geisser correction; nonparametric analyses are reported. There was a significant main effect for CP knowledge (*F* [1, 31] = 6.202, *p* = 0.018, *ƞ*^*2*^ = 0.167), a significant 3-way interaction of all the factors (*F* [1, 31] = 5.218, *p* = 0.029, *ƞ*^*2*^ = 0.144), and a significant interaction between ratio and condition (*F* [1, 31] = 4.533, *p* = 0.041, *ƞ*^*2*^ = 0.128). Other main and interaction effects were not significant (condition: *F* [1, 31] = 0.105, *p* = 0.748, *ƞ*^*2*^ = 0.003; ratio: *F* [1, 31] = 0.163, *p* = 0.689, *ƞ*^*2*^ = 0.005; condition × CP knowledge: *F* [1, 31] = 0.515, *p* = 0.478, *ƞ*^*2*^ = 0.016; ratio × CP knowledge: *F* [1, 31] = 0.924, *p* = 0.344, *ƞ*^*2*^ = 0.029). We compared the accuracy between congruent and incongruent conditions to explore the congruity effect in each condition. Fig 2 shows the congruity effect for the non-CP-knowers group for low ratio, which reached a significant level in simple effect analysis (*F* [1, 31] = 7.594, *p* = 0.01, *ƞ*^*2*^ = 0.197) and the Wilcoxon non-parametric test (*Z* = −2.016, *p* = 0.044). However, there was no significant effect for high ratio in the non-CP-knowers group (simple effect analysis: *F* [1, 31] = 0.025, *p* = 0.875, *ƞ*^*2*^ = 0.001; Wilcoxon test: *Z* = −0.09, *p* = 0.929). In the CP-knowers group, no effect was found (low ratio: simple effect analysis: *F* [1, 31] = 0.236, *p* = 0.630, *ƞ*^*2*^ = 0.008; Wilcoxon test: *Z* = −0.586, *p* = 0.558; high ratio: simple effect analysis: *F* [1, 31] = 0.801, *p* = 0.378, *ƞ*^*2*^ = 0.025; Wilcoxon test: *Z* = −0.709, *p* = 0.478). The interaction between ratio and condition corresponds to the ‘‘ratio effect” or “distance effect” [22], which is signature of the use of an ANS. The congruity effect was significant only in the low ratio using simple effect analysis (*F* [1, 31] = 5.293, *p* = 0.028, *ƞ*^*2*^ = 0.146) or the Wilcoxon test, *Z* = −1.981, *p* = 0.048). There was no significant effect in the high ratio (*F* [1, 31] = 0.273, *p* = 0.6, *ƞ*^*2*^ = 0.009; Z = −0.284, *p* = 0.776).

**Reaction time (RT) analyses**. Mean RTs and their standard deviation from all correct trials for each child were computed for each condition and task. We excluded RT data that exceeded the mean (for each participant and each task separately; fewer than 5% of trials) by more than 2 standard deviations) from the RT analyses [25]. We conducted a 2 (CP knowledge: CP knowers or non-CP knowers) × 2 (ratio: high ratio, low ratio) × 2 (condition: congruent, incongruent) ANCOVA, controlling for the RT of the color discrimination task and age in months. The Box's M test result was not significant (Box's M test = 17.988, *F* = 1.553, *p* = 0.114), which showed that the homogeneity of variance assumption was supported. The only significant 2-way interaction was of condition and CP knowledge (*F* [1, 30] = 6.042, *p* = 0.020, *ƞ*^*2*^ = 0.168). The main effects and other interactions were not significant (condition: *F* [1, 30] = 0.226, *p* = 0.638, *ƞ*^*2*^ = 0.007; ratio: *F* [1, 30] = 3.043, *p* = 0.091, *ƞ*^*2*^ = 0.092; CP knowledge: *F* [1, 30] = 0.559, *p* = 0.461, *ƞ*^*2*^ = 0.018; ratio × CP knowledge: *F* [1, 30] = 1.812, *p* = 0.188, *ƞ*^*2*^ = 0.057; ratio × condition: *F* [1, 30] = 0.406, *p* = 0.529, *ƞ*^*2*^ = 0.013). The simple effect analysis showed significant effects of condition for both CP knowers and non-CP knowers (CP: *F* [1, 30] = 9.043, *p* = 0.005, *ƞ*^*2*^ = 0.232; non-CP: *F* [1, 30] = 42.218, *p* = 0.000, *ƞ*^*2*^ = 0.585). We computed the RT-congruity effect (incongruent RTs—congruent RTs) and used t-test to compare the congruity effect between CP knowers and non-CP knowers. A larger congruity effect was found for non-CP knowers (*t* = −2.412, *df* = 32, *p* = 0.02, *d* = 0.83; CP: *M* = 80.8 ms, *SD* = 66.9 vs. non-CP: *M* = 168.3 ms, *SD* = 133.6). Mean (standard deviation) RTs of CP knowers and Non-CP knowers under congruent and incongruent condition are shown in Table 1.

**Table 1:** Mean (standard deviation) RTs in groups under congruent and incongruent condition.

	CP knowers	Non-CP knowers
Congruent trails	1057.07(319.53)	1065.50 (178.19)
Incongruent trails	1137.80(315.52)	1223.44 (223.80)

#### Effect of age and ratio

**Fig 3:**
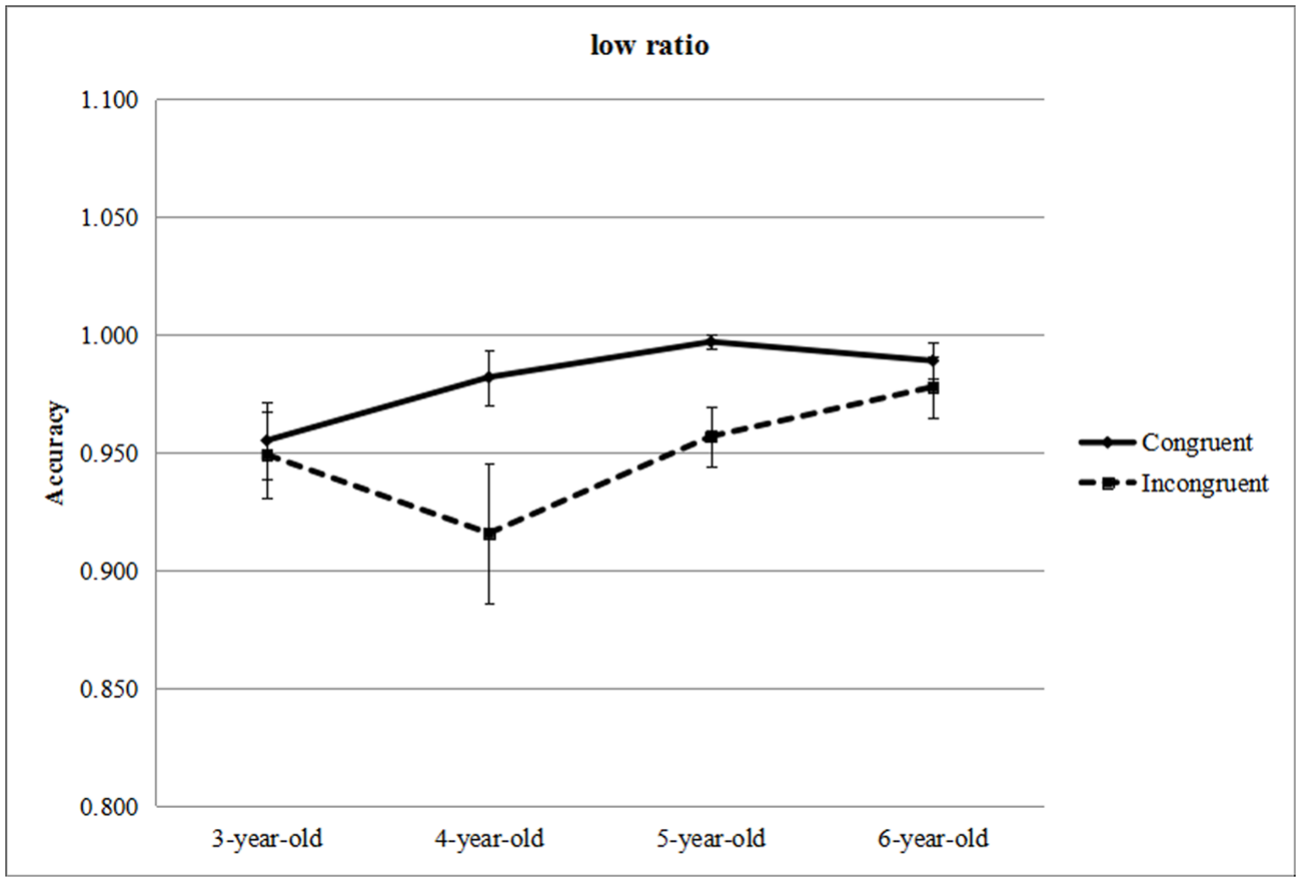
Accuracy results of interaction of age and condition in low ratio. (Error bars showed the standard errors).

**Accuracy analyses**. Accuracy (ACC) of all trials reached 97.5%. We conducted a 4 (age: 3 years, 4 years, 5 years and 6 years) × 2 (ratio: high ratio, low ratio) × 2 (condition: congruent, incongruent) analysis of repeated-measures ANOVA. The Box's M test result for the homogeneity of variance hypothesis was significant (Box's M test = 143.387, *F* = 4.284, *p* = 0.00). Greenhouse–Geisser corrections for homogeneity of variance violations were implemented. Both parametric and nonparametric analyses are reported here. The main effect for condition was significant (*F* [1, 76] = 8.155, *p* = 0.006, *ƞ*^*2*^ = 0.10), reflecting more correct responses to congruent trials (*M* = 0.98, *SD* = 0.04) compared with incongruent (*M* = 0.96, *SD* = 0.06) trials. An age effect was also present (*F* [3, 76] = 4.24, *p* = 0.008, *ƞ*^*2*^ = 0.143). There was an interaction between condition and ratio (*F* [1, 76] = 4.077, *p* = 0.047, *ƞ*^*2*^ = 0.05), as well as a significant 3-way interaction of all factors (*F* [3, 76] = 3.414, *p* = 0.022, *ƞ*^*2*^ = 0.12). Other main and interaction effects were not significant (ratio: *F* [1, 76] = 2.214, *p* = 0.141, *ƞ*^*2*^ = 0.028; ratio × age: *F* [3, 76] = 0.268, *p* = 0.849, *ƞ*^*2*^ = 0.010; condition × age: *F* [3, 76] = 0.507, *p* = 0.679, *ƞ*^*2*^ = 0.020). We also compared the accuracy between congruent and incongruent conditions to explored congruity effects in each condition. The simple effect analysis and the Wilcoxon non-parametric test revealed significant congruity effects in low ratio trials only for the 4- and 5-year-old groups (age 3: *F* [1, 76] = 0.071, *p* = 0.790, *ƞ*^*2*^ = 0.001; *Z* = −0.17, *p* = 0.865; age 4: *F* [1, 76] = 13.037, *p* = 0.001, *ƞ*^*2*^ = 0.146; *Z* = −2.58, *p* = 0.010; age 5: *F* [1, 76] = 5.621, *p* = 0.02, *ƞ*^*2*^ = 0.07; *Z* = −2.75, *p* = 0.006; age 6: *F* [1, 76] = 0.239, *p* = 0.626, *ƞ*^*2*^ = 0.003; *Z* = −0.71, *p* = 0.480). In high ratio trials there were no significant effects (age 3: *F* [1, 76] = 3.185, *p* = 0.078, *ƞ*^*2*^ = 0.04; *Z* = −1.21, *p* = 0.227; age 4: *F* [1, 76] = 0.002, *p* = 0.963, *ƞ*^*2*^ = 0.00; *Z* = −0.09, *p* = 0.929; age 5: *F* [1, 76] = 0.262, *p* = 0.61, *ƞ*^*2*^ = 0.003; *Z* = −1.00, *p* = 0.317; age 6: *F* [1, 76] = 0.118, *p* = 0.732, *ƞ*^*2*^ = 0.002; *Z* = −0.58, *p* = 0.564). We computed the size of the congruity effect based on the accuracy data (congruent—incongruent) and did not find a significant difference between 4- and 5-year-olds (*t* = 0.5, *df* = 48, *p* = 0.62, *d* = 0.14). Fig 3 and the congruity effect for each condition in Table 2 show how the effect tended to decrease. Excluding the 3-year-old children, who had not begun to show a significant congruity effect, we carried out correlational analyses between ACC-congruity effect in low ratio and months of age in the 4-, 5-, and 6-year-olds and found a significant negative correlation (*r* [65] = −0.251, *p* = 0.04). We did not find significant correlation for high ratio (*p* = 0.54).

**Table 2:** Mean accuracy and reaction times (standard deviation) under each condition.

		3 years old	4 years old	5 years old	6 years old
Low ratio	ACC				
Mean congruity effect		0.006 (0.082)	0.066 (0.121)	0.040 (0.067)	0.011 (0.062)
Congruent trials		0.955(0.062)	0.982(0.056)	0.997(0.016)	0.989(0.029)
Incongruent trials		0.949(0.071)	0.916(0.141)	0.957(0.067)	0.978(0.049)
	RT				
Mean congruity effect		133.22(228.48)	188.71(201.51)	174.96(134.84)	83.50 (85.62)
Congruent trials		1118.53(276.70)	1014.92(207.08)	850.62(199.67)	797.97(298.06)
Incongruent trials		1251.75(389.61)	1203.63(308.19)	1025.58(276.72)	881.48(299.94)
High ratio	ACC				
Mean congruity effect		0.029 (0.082)	−0.001 (0.084)	0.006 (0.03)	0.006 (0.038)
Congruent trials		0.967(0.053)	0.962(0.057)	0.994(0.022)	0.994(0.022)
Incongruent trials		0.938(0.067)	0.963(0.061)	0.988(0.030)	0.989(0.029)
	RT				
Mean congruity effect		61.34 (232.33)	115.79 (129.33)	64.846 (98.79)	106.82(117.66)
Congruent trials		1176.33(463.98)	1019.57(219.90)	890.63(222.31)	749.32(219.35)
Incongruent trials		1237.67(363.67)	1135.37(277.35)	955.47(232.75)	856.14(322.28)

ACC: accuracy; RT: reaction time.

**RT analyses**. Mean RTs from all correct trials for each child were computed. We conducted a 4 (age: 3 years, 4 years, 5 years, and 6 years) × 2 (ratio: high ratio, low ratio) × 2 (condition: congruent, incongruent) ANCOVA, controlling for the RTs of the color discrimination task. The Box's M test result for the homogeneity of variance hypothesis was significant (Box's M test = 126.05, *F* = 3.76, *p* = 0.00). Greenhouse–Geisser corrections for homogeneity of variance violations were implemented. Only condition showed a significant main effect (*F* [1, 75] = 3.976, *p* = 0.05, *ƞ*^*2*^ = 0.05). Other main and interaction effects were not significant (ratio: *F* [1, 75] = 2.286, *p* = 0.135, *ƞ*^*2*^ = 0.03; ratio × age: *F* [3, 75] = 2.037, *p* = 0.116, *ƞ*^*2*^ = 0.075; condition × age: *F* [3, 75] = 1.316, *p* = 0.276, *ƞ*^*2*^ = 0.05; condition × ratio: *F* [3, 75] = 2.437, *p* = 0.123, *ƞ*^*2*^ = 0.03; *F* [3, 75] = 1.168, *p* = 0.328, *ƞ*^*2*^ = 0.045; age: *F* [3, 76] = 1.696, *p* = 0.175, *ƞ*^*2*^ = 0.064). We used t-test analysis and the Wilcoxon non-parametric test to compare RTs between congruent and incongruent trials for 2 ratio conditions in different age groups; a significant congruity effect was found in the 3-year-old group (low ratio: *t* = 2.258, *df* = 14, *p* = 0.042, *d* = 0.39; *Z* = −2.27, *p* = 0.023; high ratio: *t* = 1.023, *df* = 14, *p* = 0.32, *d* = 0.15; *Z* = −1.59, *p* = 0.112), 4-year-old group (low ratio: *t* = 4.491, *df* = 22, *p* = 0.00, *d* = 0.72; *Z* = −3.71, *p* = 0.000; high ratio: *t* = 4.294, *df* = 22, *p* = 0.00, *d* = 0.46; *Z* = −3.41, *p* = 0.001), 5-year-old group (low ratio: *t* = 6.742, *df* = 26, *p* = 0.00, *d* = 0.73; *Z* = −4.54, *p* = 0.000; high ratio: *t* = 3.411, *df* = 26, *p* = 0.002, *d* = 0.28; *Z* = −2.811, *p* = 0.005), and 6-year-old group (low ratio: *t* = 3.777, *df* = 14, *p* = 0.002, *d* = 0.28; *Z* = −2.897, *p* = 0.004; high ratio: *t* = 3.516, *df* = 14, *p* = 0.003, *d* = 0.39; *Z* = −3.294, *p* = 0.001). We also carried out correlational analyses between RT-congruity effect and age in months for the 4-, 5-, and 6-year olds and found a significant negative correlation (*r* [65] = −0.282, *p* = 0.02).

#### Number comparison task

We used a number comparison task that could measure symbolic number discrimination abilities. We calculated the mean reaction time and correct rate for this task as an indicator of mastering symbolic number knowledge. Mean percentage of correct responses was 87% (*SD*: 0.11) and the mean reaction time was 1685.56 ± 631.13 ms. We examined the correlation between size congruency effects and numerical discrimination ability, using the accuracy and RT measurements. There was a non-significant negative correlation between the accuracy of the number comparison task and the ACC-congruency effect (*r* [40] = -0.272, *p* = 0.081), controlling for age in months. Mean RT of the number comparison task was found to significantly correlate with the RT-congruency effect (*r* [40] = 0.343, *p* = 0.026), controlling for age in months.

In summary, we found that automatic non-symbolic numerical processing began at age 3- to 4-years old and size congruity effects tended to reduce with increasing age. The study also found a CP-knowledge effect and ratio effect.

However, some researchers have suggested that inhibitory control is necessary to resolve the conflict between visual and numerical parameters [26–29]. These researchers manipulated visual cues of dots (e.g. convex hull, total surface area) and asked participants to decide which display contained more dots. In the incongruent trials, the visual characteristics and numerosity were inconsistent, when there were more dots and smaller visual properties. The researchers believed that participants needed to inhibit the incongruent visual characteristics of an array and focus on numerosity. Participants with better inhibitory control skills were likely to perform better at dot comparison task trials that contained incongruent visual cue information. Therefore, we also need to be aware of the impact of inhibition skills in the size congruity task. Completing this task requires inhibition of inconsistent numerosity information. The size congruity effect is likely to be related to inhibition skills.

To directly test this hypothesis we conducted a second experiment in which we measured inhibition skill in 4-year-old children with stable size congruity effects. Additionally, we were also concerned about the interference and facilitatory components of the size congruity effect by using neutral condition (the number of dots is the same but the physical size differs). In the congruent trials, the RTs were shorter and accuracy was higher than in the neutral trials (facilitation effect); whereas in the incongruent trials, the RTs were longer and the accuracy lower than in the neutral trials (interference effect).

## Experiment 2

### Methods

#### Participants

A total of 31 children participated in the study (mean age ± SD: 49.93 ± 2.03 months; range: 46–54 months; 15 boys), all from the same kindergarten as in Experiment 1.

#### Day-night task

Inhibition was assessed using the day-night task [30]. In the task, children were instructed to say “night” when presented with a picture of a yellow sun in a white sky and “day” when presented with a picture of a yellow moon in a dark sky. Each condition started with 4 training trials with feedback for each trial, followed by 16 test trials. Trials were presented in a fixed pseudorandom order with the constraint that the same picture never appeared in 3 consecutive trials. Each correct response was credited with 1 point.

#### Size congruity task

Tasks and stimuli were the same as in Experiment 1, except we added neutral trials to measure the facilitation and interference effects. A total of 68 trials were administered to each participant: 8 practice trials, 24 incongruent, 24 congruent, and 12 neutral trials (see Appendix). In the neutral condition 2 arrays containing the same number of dots were presented. The size of dots in one group was larger (radius: 0.9 cm) than the other (radius: 0.5 cm), similarly to Experiment 1.

### Results

#### Day-night task

Pearson correlations were calculated between day-night scores and the congruity, facilitation, and interference effects, and accuracy and RTs in each condition (*ps*>0.05, Table 3). However, none of the correlations were significant, confirming independence between the development of inhibition and automatic numerical processing.

**Table 3:** Correlations between day-night scores and congruity, facilitation, and interference effects, and accuracy and RTs in each condition.

	ACC-congruity effect (congruent—incongruent)	RT-congruity effect (incongruent—congruent)	ACC-facilitation effect (congruent—neutral)	ACC-interference effect (neutral—incongruent)
Day-night scores	−0.258 (*p* = 0.161)	0.308 (*p* = 0.092)	−0.083 (*p* = 0.658)	−0.159 (*p* = 0.393)
	ACC-congruent	ACC-incongruent	ACC-neutral	RT-congruent
Day-night scores	0.098 (*p* = 0.602)	0.287 (*p* = 0.118)	−0.247 (*p* = 0.180)	−0.326 (*p* = 0.073)
	RT-facilitation effect (neutral—congruent)	RT-interference effect (incongruent—neutral)	RT-incongruent	RT-neutral
Day-night scores	0.195 (*p* = 0.294)	0.149 (*p* = 0.423)	−0.258 (*p* = 0.171)	−0.247 (*p* = 0.180)

The p-values of the correlations between day and night scores and the RT congruity effect, day and night scores and the RT-congruent, as well as day and night scores and the ACC-incongruent were marginal significant. We added bootstrapped 95% confidence intervals (1,000 bootstrapping samples) and calculated Bayes factors for the five largest correlation scores to verify this result. The results showed in the Table 4. The Bayes factors that between 1/3 to 3 showed that we did not have enough evidence to prove whether the correlations exist.

**Table 4:** 95% confidence intervals and Bayes factors.

	RT congruity effect	RT-congruent	ACC-incongruent	ACC-congruity effect	RT-incongruent
confidence intervals	0.28 to 0.34	-0.38 to -0.26	0.26 to 0.32	-0.32 to -0.19	-0.32 to -0.19
Bayes factors	B = 1.05	B = 1.22	B = 0.90	B = 0.73	B = 0.49

#### ACC analyses

ACC of all trials reached 96.5%. A 3-way analysis of repeated measures ANOVA (conditions: congruent, neutral, incongruent) was conducted. Greenhouse–Geisser corrections for homogeneity of variance violations were implemented as determined by Mauchly’s test. The main effect was significant (*F* [2, 29] = 11.394, *p* = 0.00, *ƞ*^*2*^ = 0.275; Friedman test: *χ*^*2*^ = 17.422, *df* = 2, *p* = 0.000). A post-hoc LSD test indicated a significant difference between the congruent and incongruent condition (*p* = 0.000; *M* = 0.982, *SD* = 0.06 vs. *M* = 0.936, *SD* = 0.012) as well as the incongruent and neutral condition (*p* = 0.003; *M* = 0.982, *SD* = 0.06 vs. *M* = 0.978, *SD* = 0.007). The difference between the congruent and neutral condition was not significant (*p* = 0.632). The same results in the Wilcoxon test (*Z* = −3.559, *p* = 0.000; *Z* = −3.001, *p* = 0.003; *Z* = −0.601, *p* = 0.548).

We conducted a 3-way analysis of repeated-measures ANOVA (condition: incongruent in low ratio, neutral, incongruent in high ratio). Mauchly's test of sphericity showed that homogeneity of variance was not violated. The main effect was significant (*F* [2, 29] = 6.749, *p* = 0.004, *ƞ*^*2*^ = 0.318). A post-hoc LSD test indicated a significant difference between the incongruent condition in low ratio and the neutral condition (*p* = 0.001; *M* = 0.930, *SD* = 0.012 vs. *M* = 0.978, *SD* = 0.007). The difference between the incongruent condition in high ratio and the neutral condition (*p* = 0.061; *M* = 0.943, *SD* = 0.017 vs. *M* = 0.978, *SD* = 0.007) and between the incongruent condition in high ratio and in low ratio (*p* = 0.492) was not significant.

#### RT analyses

Mean RTs from all correct trials were computed. Controlling for the RTs of the color discrimination task, a 3-way analysis of repeated-measures ANCOVA (conditions: congruent, neutral, incongruent) was conducted. Mauchly's test of sphericity showed that homogeneity of variance was not violated. The main effect was significant (*F* [2, 28] = 7.618, *p* = 0.002, *ƞ*^*2*^ = 0.352). A post-hoc LSD test indicated a significant difference between the congruent and incongruent condition (*p* = 0.000; *M* = 1119.344, *SD* = 42.869 vs. *M* = 1020.976, *SD* = 34.743) as well as the incongruent and neutral condition (*p* = 0.000; *M* = 1119.344, *SD* = 42.869 vs. *M* = 1046.336, *SD* = 35.228). The difference between the congruent and neutral condition was not significant.

We conducted a 3-way (conditions: incongruent in low ratio, neutral, incongruent in high ratio) analysis of repeated-measures ANCOVA, controlling for the RTs of the color discrimination task. Mauchly's test of sphericity showed that homogeneity of variance was not violated. The main effect was significant (*F* [2, 28] = 11.060, *p* = 0.000, *ƞ*^*2*^ = 0.441). A post-hoc LSD test indicated a significant difference between the incongruent condition in low ratio and the neutral condition (*p* = 0.000; *M* = 1152.975, *SD* = 45.699 vs. *M* = 1046.336, *SD* = 35.228), between the incongruent condition in high ratio and the neutral condition (*p* = 0.038; *M* = 1085.713, *SD* = 42.837 vs. *M* = 1046.336, *SD* = 35.228), and the incongruent condition in high ratio and in low ratio (*p* = 0.005).

We computed the RTs-interference effect (incongruent RTs—neutral RTs) respectively in the high ratio and low ratio conditions and used t-test to compare the effect. A larger RTs-interference effect was found for the low ratio condition (*t* = 2.684, *df* = 30, *p* = 0.012, *d* = 0.53; low ratio: *M* = 106.64, *SD* = 128.13 VS high ratio: *M* = 39.38, *SD* = 127.40).

## Discussion

We studied the development of automated non-symbolic magnitude and its influencing factors. We first explored the development of the size congruity effect in 3- to 6-year-old children. Second, whether the mastery of CP knowledge and number comparison ability related to the size congruity effect in children was investigated. Third, we questioned whether ratio affected the automatic processing of non-symbolic magnitude. Finally, we explored the relationship between the size congruity effect and inhibition skill, as well as interference and facilitatory components in children aged 4 years old.

Our experiment documented that automatic access to non-symbolic magnitude began at 3- to 4-years-old and size congruity effects tended to reduce with increasing age from 4 years old. This developmental trend is in agreement with previous research in school children [13]. In addition, we found a congruity effect in 3-, 4-, 5-, and 6-year-olds, in accordance with similar findings in previous research [1, 14]. Three-year-old children demonstrated a congruity effect with RTs only in a low ratio, indicating that the automated processing of 3-year-old children is not stable. In contrast to our results, Rousselle and Noel (2008) found that the existence of automatic access began at age 3 years and the congruity effect tended to increase with age [14]. This inconsistency in results may be due to different task and stimulus materials. In their task, children were asked to compare total filled areas, encouraging in-depth processing and possibly facilitating the processing of magnitude information. Area as a continuous variable can affect the numerosity judgment of young children [31–34]. As a result, a question about areas may increase the activation of numerical processing. Besides, Odic, Libertus, Feigenson, and Halberda (2013) also found that both area and number discrimination obeyed Weber’s law and area acuity steadily improved in early childhood [35]. Developmental improvements in area discrimination may make it easier for older children to perform Rousselle and Noel’s task. This may result in greater automatic activation of numerical processing in older children. Another possible factor is that they used 2 ratios (1:2, 2:3) which are higher than our low ratios (0.11–0.33) but lower than the highest ratios (0.67–0.89). Future research can further compare the two tasks.

It may also be necessary to consider the role of inhibitory mechanisms in automatic processing; however, previous research suggests the development of inhibiting ability is independent of automatic numerical processing [14]. The p-value of our findings supported this view: the results of p-value showed no significant correlation between inhibition skills and size congruity effect. Some research has proposed that inhibition ability is necessary to resolve the conflict between visual and numerical parameters [26–29]. The contrasting results may be explained by the different nature of the tasks. In their dot comparison tasks, the task requirement was the identification of the quantity of dots. Numerosity is an abstract parameter, different from visual characteristics. Participants need to extract numerical information from the non-symbolic stimulus defined by visual properties. Therefore, inhibiting visual information during numerical processing may be more difficult than inhibiting numerical information during perceptual processing. In our size congruity task, completing perceptual discrimination required less inhibition ability. This is probably why inhibition ability does not relate to the size congruity effect. However, it should be noted that this result is not stable: the Bayes factors showed that the result could not be considered as a substantial evidence. The unstable results might result from the limitation of sample size: our sample size just met the minimum requirements for correlation analysis. So, to address this issue, further exploration with a larger sample size is needed.

We investigated the CP-knowledge effect. Negen and Sarnecka (2015) found that children at the non-CP-knower level performed by chance in numerosity comparison tasks (standard ANS task), indicating that they may not have understood the task [36]. Studies have also shown that young children had difficulty in numerical comparison when perceptual features such as surface area were in conflict with numerosity [29, 32]. These results remind us that young children needed to understand our task, which required participants to choose the physically larger group, in which there was a larger total surface area and all dots the same size. This task is easier than the numerosity comparison tasks in the previous study (e.g. Merkley et al., 2015) [29]; additionally, we gave feedback to the participants in the practice trials to ensure that they indeed understood the task. In our results, the lowest mean accuracy for non-CP knowers was 0.88 in the low ratio incongruent condition. Therefore, we believe that participants fully understand the task instructions.

Our study found that CP knowledge had relationship with the size congruity effect and the non-CP knowers had a larger congruity effect with low ratio. In addition, the ability to process symbolic magnitude was negatively correlated with congruity effect. These findings suggest developmental change occurs and support the hypothesis that increased utilization of symbolic numerosity processing may result in the reduction of non-symbolic size congruity effects, which index the ability to automatically process non-symbolic numerosities. It is well-known that training and practice can enhance automatized performance in different cognitive processing [37, 38]. Research has also found that practicing a dimension increases its interference effect when the dimension is task-irrelevant, in the Stroop task [38]. Similarly, the automatic processing of number (size congruity effect) is also achieved gradually as children’s numerical skills and knowledge progress, which could account for the development of automatic symbolic numerosity processing [5, 12]. The role of practice is not only reflected in the production of automaticity of new skills, but also reflected in the reduction of old skills. The researchers proposed that lack of practice could explain the reduction of automatic non-symbolic numerosity processing with age [13]. After 4 years old, children had a large change that they began to grasp the symbolic number. The developmental change in our findings might result from the utilization of non-symbolic representation that is reducing with age. Cohen, Berger, Rubinsten, and Henik (2014) found that children who learned a new symbolic set of numerals showed size congruity effects in the new symbolic system and no effect for the originally learned symbolic set [39]. Some transfer between the 2 symbolic systems may occur. In the development of the automatic processing of numerosity, the non-symbolic and symbolic system may also similarly transfer after 4 years old, resulting from the reduction of utilization of non-symbolic representation and an increase in utilization of symbolic representation. Despite the lack of a causal relationship, the finding provides worthy evidence of the relationship between symbolic and non-symbolic numerosity processing.

We also found the size congruity effect more easily and a larger interference effect in low ratio conditions, providing evidence that children used approximate number representation when performing the physical size comparisons task. These results add to a growing body of evidence that ratio or distance effects exist in automatic numerical processing and are consistent with previous reports on both symbolic and non-symbolic stimuli in school children and adults [6, 13, 22, 23, 40]. A similar pattern was found in the neural mechanisms underlying the development of automated magnitude: the onset of stimulus-locked lateralized readiness potentials (SLRP) was delayed for large-distance compared with small-distance trials with school children and adults [13, 23]. Additionally, in number—size congruity tasks, close distance trials (compared with far ones) yielded significant activations in middle and inferior frontal brain regions (including the dorsolateral prefrontal cortex) on the right in 8- to 12-year-old children [41].

However, not all studies have shown ratio/distance effect in automatic processing of number magnitude information [12, 42]. Tzelgov et al. (1992) asked adults to judge if the given number was smaller or larger than the criterion stated and found the numerical distance between 2 digits influenced performance only in numerical judgments and was absent in physical judgments [42]. They proposed that autonomous activation of numerical information mainly resulted in the classification of numerical stimuli as large or small and digits larger or smaller than 5 were classified as “large” or “small”. This processing was mainly memory based. Using the number Stroop task (i.e., comparing 2 numbers’ physical/numerical size presented on the screen), Rubinsten, Henik, Berger, and Shahar-Shalev (2002) also did not find the numerical distance effect for physical size judgments [12]. They proposed a 2-representation model of numerical magnitude processing: an algorithmic-based process which maps digits onto an analogical number line in an intentional process and a memory-based process which classify numerals as large or small in an autonomous process.

On the contrary, these results are inconsistent with Dehaene and Akhavein’s (1995) model of single representations of number magnitude [43]. They found a distance effect when the same-different task did not require the use of numerical values, suggesting that magnitude information was automatically processed. Jiang et al. (2015) supported the single-representation model and thought that the use of an asymmetrical design and the potential interactions between congruity and numerical distance might have contributed to the results of Rubinsten et al (2002) [44]. They found no dissociation between congruity and numerical distance with a symmetrical design manipulating physical size distance systematically, a positive distance effect under the congruent condition, and a negative distance effect under the incongruent condition in the physical judgment task.

In the present study, we also found the size congruity effect more easily in low ratio conditions. Our non-symbolic task was different from Tzelgov et al. (1992) and Rubinsten et al. (2002), with the number of dots larger than 5. The classification of digits as large or small was mainly memory-based, as the previous studies mentioned. In the non-symbolic numerosity task, because of imprecision of large numerical magnitude representation, subjects had difficulty retrieving from memory to judge the whether the number of dots was large or small. Therefore, our finding of a ratio effect supports an algorithmic-based process which maps magnitude onto an analogical number line, at least in automatic non-symbolic large numerosity processing.

The ratio effect finding suggested that subjects detected a smaller difference between 2 non-symbolic stimuli with greater difficultly, resulting in less numerosity activation in the high ratio. Children responded more correctly in the incongruent trials of low ratio than in neutral trials (ratio: 1). Children responded more slowly in the incongruent trials of low ratio than in high ratio, and more sequentially than in neutral trials. The larger interference effect in the low ratio condition indicated that the precision of discriminating the numerical difference between 2 sets of dots decreased with the ratio. Thus, young children might use approximate number representation when automatically processing large numerical magnitude. Because the ANS is active early in infancy, children mostly rely on it before learning symbolic representation, when representing large cardinal values [19]. Preschool children reasonably automatically process non-symbolic large numerosities with the ANS. Our results provided evidence in favor of the ratio or distance effect in automaticity of number magnitude processing.

Our results demonstrated the presence of a significant interference effect but no facilitation effect for non-symbolic processing in 4-year-old children. The finding was consistent with Rousselle and Noël (2008) who also only found an interference effect in non-symbolic processing in 4- to 6-year olds [14], while Gebuis et al. (2009) found both interference and facilitation effects in 5-year-olds [1]. Further, the interference effect in children was larger than the facilitation effect [1]. Gebuis et al. (2009) also found that second graders demonstrated both facilitation and interference effects for a non-symbolic task [13]. It appears that the facilitation effect was not steady. In a symbolic task, there were also similar results. In symbolic Size Congruity Task, participants must choose the physically larger number (e.g. 2 5) and ignore a task-irrelevant dimension (the numerical magnitude). As automatic processes, automatic processing of symbolic and non-symbolic numerical magnitude have domain-general features. Zhou et al. (2007) found only an interference effect in 5-year-old Chinese children [15]. Some research has shown that only interference effects exist in 6- to 8-year olds [5, 12, 13], while Bugden and Ansari (2011) found both interference and facilitation effect in Grade 1 (7-year-old) children [6]. The finding of an unstable facilitation effect is consistent with previous studies using another Stroop task. The interference effect in the Stroop task is known to be strong and stable, while the facilitation effect is smaller and less stable, and may sometimes be absent [45–46].

To conclude, this study traced the development and explored the influencing factors of automatic numerical processing of non-symbolic stimuli in preschool children. By the age of 3 to 4 years, children can automatically process numerosities and sensitivity to numerical cues decreases with age. The ratio effect and a larger interference effect in a low ratio suggested that the ANS plays a role in non-symbolic physical size comparison tasks. The effect of CP knowledge and its relationship with number comparison tasks indicated that the increased utilization of symbolic representation may have a negative correlation with automatic non-symbolic numerical processing. Only significant interference effects were present in 4-year-old children. The correlation between inhibition skills and size congruity effect need further study with a larger sample.

## Supporting information

S1 Table Appendix: The materials of incongruent and congruent trials. (DOCX)

S1 File The data of experiment1. (XLS)

S2 File The data of experiment2. (XLS)
