# Supplementary material for: Automatic non-symbolic numerosity processing in preschoolers
Source: PLoS One. 2017 Jun 23;12(6):e0178396. doi: 10.1371/journal.pone.0178396 (PMC5482442; doi:10.1371/journal.pone.0178396)
Supplement: S1 Table — (DOCX) [file pone.0178396.s001.docx]

**Appendix:The materials of incongruent and congruent trials.**

| Condition | Pairs | Ratio | Times repeated |
| --- | --- | --- | --- |
| practice | 9-24 | 0.375 | 2 |
| practice | 27-33 | 0.82 | 2 |
| practice | 12-32 | 0.375 | 2 |
| practice | 8-10 | 0.8 | 2 |
| congruent | 13-35 | 0.371 | 2 |
| congruent | 6-16 | 0.375 | 2 |
| congruent | 11-29 | 0.379 | 2 |
| congruent | 10-26 | 0.385 | 2 |
| congruent | 12-31 | 0.387 | 2 |
| congruent | 7-18 | 0.39 | 2 |
| incongruent | 13-35 | 0.371 | 2 |
| incongruent | 6-16 | 0.375 | 2 |
| incongruent | 11-29 | 0.379 | 2 |
| incongruent | 10-26 | 0.385 | 2 |
| incongruent | 12-31 | 0.387 | 2 |
| incongruent | 7-18 | 0.39 | 2 |
| congruent | 7–9 | 0.78 | 2 |
| congruent | 16–20 | 0.80 | 2 |
| congruent | 20–24 | 0.83 | 2 |
| congruent | 13–15 | 0.87 | 2 |
| congruent | 28–32 | 0.88 | 2 |
| congruent | 32–36 | 0.89 | 2 |
| incongruent | 7–9 | 0.78 | 2 |
| incongruent | 16–20 | 0.80 | 2 |
| incongruent | 20–24 | 0.83 | 2 |
| incongruent | 13–15 | 0.87 | 2 |
| incongruent | 28–32 | 0.88 | 2 |
| incongruent | 32–36 | 0.89 | 2 |
| **Experiment 2** |  |  |  |
| neutral | 20-20 | 1 | 2 |
| neutral | 13-13 | 1 | 2 |
| neutral | 32-32 | 1 | 2 |
| neutral | 16-16 | 1 | 2 |
| neutral | 28-28 | 1 | 2 |
| neutral | 6-6 | 1 | 2 |
